# Multiscale simulations of uni-polar hole transport in (In,Ga)N quantum well systems

**DOI:** 10.1007/s11082-022-03752-2

**Published:** 2022-06-06

**Authors:** Michael O’Donovan, Patricio Farrell, Timo Streckenbach, Thomas Koprucki, Stefan Schulz

**Affiliations:** 1 Tyndall National Institute, Universite College Cork, Cork, T12 R5CP Ireland; 2 Department of Physics, University College Cork, Cork, T12 YN60 Ireland; 3 Weierstrass Institute (WIAS), Mohrenstr. 39, 10117 Berlin, Germany

**Keywords:** III-nitride, Charge transport, Multiscale modelling, I–V characteristics, InGaN, Fluctuations, Numerical simulation, Semiconductor device models

## Abstract

Understanding the impact of the alloy micro-structure on carrier transport becomes important when designing III-nitride-based light emitting diode (LED) structures. In this work, we study the impact of alloy fluctuations on the hole carrier transport in (In,Ga)N single and multi-quantum well systems. To disentangle hole transport from electron transport and carrier recombination processes, we focus our attention on uni-polar (*p*-*i*-*p*) systems. The calculations employ our recently established multi-scale simulation framework that connects atomistic tight-binding theory with a macroscale drift-diffusion model. In addition to alloy fluctuations, we pay special attention to the impact of quantum corrections on hole transport. Our calculations indicate that results from a virtual crystal approximation present an upper limit for the hole transport in a *p*-*i*-*p* structure in terms of the current-voltage characteristics. Thus we find that alloy fluctuations can have a detrimental effect on hole transport in (In,Ga)N quantum well systems, in contrast to uni-polar electron transport. However, our studies also reveal that the magnitude by which the random alloy results deviate from virtual crystal approximation data depends on several factors, e.g. how quantum corrections are treated in the transport calculations.

## Introduction

The semiconductor alloy indium gallium nitride ((In,Ga)N) has attracted significant research interest for optoelectronic device applications due to its in principle flexible band gap engineering across the visible spectral range (Humphreys 2008). In general, (In,Ga) N alloys have several unique features which are not found in other III–V material systems (e.g. (In,Ga)As). Firstly, heterostructures such as (In,Ga)N/GaN quantum wells (QWs) grown along the wurtzite *c*-axis exhibit strong internal electrostatic built-in fields across the QW (Ambacher et al. 2002; Caro et al. 2013). Such fields are absent in (In,Ga) As/GaAs wells grown along the [001]-direction of their underlying zincblende structures. The built-in field in *c*-plane (In,Ga)N/GaN QWs is induced by spontaneous polarization, as well as a strain related piezoelectric contribution (Ambacher et al. 2002; Caro et al. 2013). A consequence of this internal electric field is (i) a decrease in electron-hole wavefunction overlap and (ii) a red-shift in the emission wavelength; this is also known as the quantum confined Stark effect (Williams et al. 2009). Secondly, and equally important for this study, (In,Ga)N alloys and connected heterostructures display strong carrier localization effects even for a random alloy micro-structure (Watson-Parris et al. 2011; Schulz et al. 2015; Tanner et al. 2020). This effect is particularly strong for holes, which have a higher effective mass than the electrons (Schulz et al. 2015).

As such, understanding the impact of the alloy fluctuations on carrier transport becomes important when designing (In,Ga)N-based light emitting diode (LED) structures. In order to gain insight into the connection between alloy fluctuations and carrier (electron and hole) transport in (In,Ga)N-based multi-quantum well (MQW) systems, studying the properties of *uni-polar* structures present a very promising and interesting alternative to investigating a full LED structure. Previous works have focused already on uni-polar electron transport in *n*-doped-intrinsic-*n*-doped (*n*-*i*-*n*) (In,Ga)N/GaN MQW systems (Browne et al. 2015; O’Donovan et al. 2021a). These investigations revealed that alloy fluctuations (Browne et al. 2015; O’Donovan et al. 2021b) as well as quantum effects (O’Donovan et al. 2021b) are important for describing the electron transport, leading for instance to a lower knee/turn-on voltage of the device and an improved theory experiment comparison for such systems. However, far less attention has been directed towards uni-polar *hole* transport (Shen et al. 2021). Recent work has investigated uni-polar hole transport through an *(Al,Ga)N barrier* (Qwah et al. 2020). The study showed the importance of considering alloy fluctuations for the theoretical description of the hole transport in such systems. However, a similar investigation for (In,Ga)N *quantum well* systems is missing. This stems in part from the fact that high quality *p*-doped-intrinsic-*p*-doped (*p*-*i*-*p*) systems are challenging to realise experimentally (high dopant activation energy (Kozodoy et al. 2000), compensation effect (Iida et al. 2010), memory effect (Ohba and Hatano 1994)), but also from the fact that the theoretical modelling of carrier localization in (In,Ga)N systems is a difficult task in itself (Di Vito et al. 2020; Chaudhuri et al. 2021). Moreover, given the difference in the material system ((Al,Ga)N vs. (In,Ga)N) the results from Qwah et al. (2020) cannot necessarily be carried over to an (In,Ga)N/GaN structure. Therefore we focus in this work on uni-polar hole transport in (In,Ga)N QWs, with holes travelling through wells rather than barriers.

Here, we apply our previously established multi-scale simulation framework (O’Donovan et al. 2021b), that bridges the gap between atomistic electronic structure theory and macroscale drift-diffusion (DD) carrier transport simulations, to study uni-polar hole transport in (In,Ga)N single QW (SQW) and MQW systems. We analyze in detail the impact of alloy and quantum corrections on the results. Our calculations reveal that in contrast to previously reported uni-polar *electron* transport results, alloy fluctuations have a detrimental effect on the hole transport in (In,Ga)N MQWs.

The manuscript is organized as follows: In Sect. 2 we outline the theoretical framework we use, introducing the underlying tight-binding (TB) model as well as localization landscape theory (LLT) and the DD settings. In Sect. 3 we present our results for uni-polar hole transport in (In,Ga)N/GaN SQW and MQW systems. Finally, Sect. 4 concludes this work.

## Theoretical framework

In this section we outline briefly the main ingredients of our theoretical framework. A detailed discussion is given in O’Donovan et al. (2021b). In Sect. 2.1 we introduce the TB model and a “local” TB Hamiltonian that is used to obtain the local band edge energies. We then describe briefly in Sect. 2.2 how the band edge energy is transferred and connected to the device simulation mesh used in the DD solver. The DD model underlying the calculations is presented in Sect. 2.3.

### Tight-binding model and energy landscape generation

The theoretical framework starts with an $sp^3$ nearest-neighbour TB model which is described in detail in Caro et al. (2013) and Schulz et al. (2015). This approach, combined with valence force field and local polarization models, allows us to capture the impact of (random) alloy fluctuations on the electronic structure of (In,Ga)N QW systems on an atomistic scale. While it is possible to use such an atomistic electronic structure theory as the backbone for carrier transport calculations (O’Donovan 2021a; Geng et al. 2018), it is computationally very expensive to simulate a full device structure. To reduce the computational load, while still keeping essential atomistic information, we proceed as follows. In a first step, we extract an energy landscape from TB which can be used in the active region of a device. Since we are interested in uni-polar hole transport, our active region consists of (In,Ga)N QWs; in a full LED structure, the active region may also include an (Al,Ga)N electron blocking layer (EBL) (Yan Zhang and An Yin 2011). While such an EBL can also affect the hole transport, the aim of this study is *not* to investigate a full device or compare results directly with experimental data on LED structures. We are aiming for a comparative analysis that in general sheds light on different (microscopic) aspects that can affect the hole transport. Macroscopic factors such as the built-in field variations due to an EBL, which will also be affected by the distance of the EBL to the “active” region, are beyond the scope of our study. Without such an EBL, outside the active QW region a coarser mesh resolution, as described in more detail below, is used. This is motivated by the fact that the barrier regions in our case here are made up of binary GaN, which does not exhibit alloy fluctuations. In order to extract an energy landscape, we construct a “local” TB Hamiltonian from the full TB Hamiltonian, and diagonalize it at each lattice site of the simulation cell that describes the active region. In doing so one can derive a local valence (or conduction) band edge energy which contains (local) strain and polarization effects arising from alloy fluctuations. More details on the method are given in Chaudhuri et al. (2021). Equipped with such an energy landscape, either electronic structure or carrier transport calculations can be performed using modified continuum-based models (Chaudhuri et al. 2021; O’Donovan et al. 2021b).

### Device mesh generation, smoothing alloy fluctuations and quantum corrections

In this subsection, we discuss in detail key aspects of our approach to connect the TB energy landscape to DD simulations. First, we describe the device mesh structure. At its core lie two different types of meshes: an atomistic and a significantly coarser macroscopic mesh. The former corresponds to the QW/active region which the latter embeds into a device. In the following, we detail different types of smoothing operations on the atomistic mesh. We smooth the atomistic valence band edge (VBE) data obtained from TB either via Gaussian averaging, LLT or a combination of both operations. Gaussian averaging and LLT help to account for quantum effects which classical DD theory does not directly consider. In the final subsection, we pay particular attention to subtleties of applying LLT in a MQW case.

#### Device mesh structure

Our device mesh consists of an atomistic and macroscopic part. The atomistic mesh corresponds to the single or multi-well quantum region. Since we will solve the LLT equation on this mesh via finite element method (FEM), see below, we refer to it as FEM mesh as well. In this mesh, each node location and data site correspond to the position of an atom and its VBE energy, respectively. Since our goal is to study macroscopic DD currents, we embed the atomistic mesh into a macroscale device mesh with doped contact regions on either side. Our aim has two immediate implications. Given that the doped regions are a couple of orders of magnitude larger than the QW region and do not exhibit alloy fluctuations, the mesh in these regions can be chosen to be significantly coarser; this helps to reduce the computational cost. Moreover, DD simulations are typically performed via the finite volume method (FVM). Here, in particular, we use the Voronoi FVM (Farrell et al. 2017). Since this method requires a boundary-conforming tetrahedral Delaunay mesh, we not only enlarge the QW mesh by introducing meshes for the doped regions but also insert a few additional points within the QW region itself. The atomistic VBE data is then interpolated onto these additional nodes. Within the doped regions on either side of the QW region, we set uniform (GaN) VBE data. All atomistic nodes within the FEM mesh are also included in the FVM mesh. Both meshes are created via TetGen (Si 2015) and the interpolation is handled via WIAS-pdelib (Fuhrmann et al. 2019). The device mesh generation is explained visually and in more detail in Ref. O’Donovan et al. (2021).

#### Smoothing by Gaussian averaging

Previously it has been discussed that the spatial scale over which alloy fluctuations are relevant for carrier transport is linked to the de Broglie wavelength of the carriers (Li et al. 2017). Given the semi-classical and continuum-based nature of “standard” DD models, such effects are not captured. To remedy this shortcoming, in a first step we employ a Gaussian averaging procedure on the FEM mesh given by

1 $$\begin{aligned} E_{v}^{\sigma }\left( \mathbf {x_i}\right) = \frac{\sum _j E_{v}^\text {TB}\left( \mathbf {x_j}\right) \times \exp \left( \frac{-\Vert \mathbf {x_i}-\mathbf {x_j}\Vert ^2}{2\sigma ^2}\right) }{\sum _j \exp \left( \frac{-\Vert \mathbf {x_i}-\mathbf {x_j}\Vert ^2}{2\sigma ^2}\right) }\,\, . \end{aligned}$$

The averaging procedure accounts now for the effect that carrier wavefunctions do not only “see” valence band energies at a given lattice site but also beyond this. In doing so, the averaging procedure depends on the width of the Gaussian, $\sigma$. We note that the above is similar to Li et al. (2017), however our approach differs in that we average the TB band edge energy, $E_{v}^\text {TB}$, which contains already local strain and built-in polarization field effects obtained on an atomistic level; in Li et al. (2017) first local In, Ga contents are determined and then, using continuum elasticity theory, local strain and built-in polarization potentials are evaluated before the local band edge energy values are calculated. Given that the Gaussian width $\sigma$ is now effectively a free parameter, we will study below the impact of $\sigma$ on the effective energy landscape and the hole transport. Future studies may target evaluating $\sigma$ values based on calculations of e.g. the density of states (Piccardo et al. 2017; McMahon et al. 2020) in (In,Ga)N-based QWs utilizing modified continuum models.

**Fig. 1:**
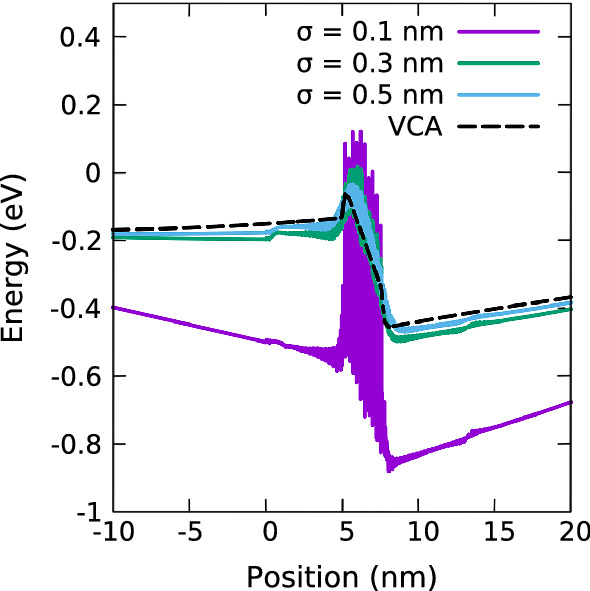
Comparison of valence band edge energies for an In$_{0.1}$Ga$_{0.9}$N single quantum well of width 3.1 nm at a bias of 0 V (equilibrium solution) without quantum corrections for a VCA (black, dashed) and random alloy calculations using a Gaussian width, $\sigma$, of 0.1 nm (purple), 0.3 nm (green) and 0.5 nm (blue). (Color figure online)

To understand the potential impact of $\sigma$ on the results, Fig. 1 shows the VBE energy profile of an (In,Ga)N/GaN SQW with 10% In and a width of 3.1 nm for different values of $\sigma$ ($\sigma = 0.1$ nm (purple), $\sigma = 0.3$ nm (green) and $\sigma = 0.5$ nm (blue)) at equilibrium (0 V). The VCA profile, which does not undergo broadening, is also depicted (black, dashed). Firstly, we note that when choosing a $\sigma$ value smaller than the bond length of the material, $d_0$ (e.g. $\sigma = 0.1$ nm $< d_0^\text {GaN}$ (Tanner 2017)), basically no averaging takes place. As a consequence, the VBE energy exhibits very strong fluctuations due to the alloy fluctuations, see Fig. 1. We note that while the average QW “depth” (averaged over each atomic plane) does not differ significantly for different $\sigma$ values, both the magnitude of the VBE energy fluctuations as well as the potential barrier between (In,Ga)N well and surrounding GaN is strongly impacted by the $\sigma$ value. Thus, Fig. 1 gives already indications that carrier transport, e.g. current voltage (I–V) curves, may be strongly dependant on $\sigma$. We will discuss this in more detail below.

#### Quantum corrections by localization landscape theory

While the above introduced Gaussian averaging procedure provides local corrections to the confining energy landscape, it does not provide information about the electron and hole ground state energy in a QW system since it is not coupled with a quantum mechanical description by e.g. solving the Schrödinger equation. On the other hand, most conventional/commercial transport simulators often have the option to couple DD simulations with solving Schrödinger’s equation, however, they neglect alloy fluctuations. As discussed for instance in detail in Li et al. (2017), it is numerically very demanding to study carrier transport in (In,Ga)N/GaN LED structures when treating alloy fluctuations and quantum corrections in a fully 3-D self-consistent Schrödinger-Poisson-DD framework. To this end we employ the numerically far more efficient localization landscape theory (LLT) (Arnold et al. 2016; Filoche et al. 2017; Chaudhuri et al. 2020) to account for quantum corrections in our 3-D simulations. Thus instead of solving Schrödinger’s equation, we solve the LLT equation supplied with Dirichlet and Neumann boundary conditions:

2 $$\begin{aligned} \hat{H}^\text {EMA}u = \left( -\frac{\hbar ^2}{2m^\star }\Delta + (V - E_\text {ref})\right) u = 1\,\, . \end{aligned}$$

Here $m^\star$ denotes the effective mass, $\hbar$ is Planck’s constant, and $E_\text {ref}$ is the reference energy of the system. The choice of $E_\text {ref}$ will be discussed in detail in Sect. 2.2.4. *V* is the confining potential which is extracted from the local band edge energy values: since we are targeting uni-polar hole transport, *V* is determined by the VBE energy.^1^

We note that LLT involves solving a linear partial differential equation instead of a large eigenvalue problem as in case of the Schrödinger equation. Therefore, LLT facilitates a numerically more efficient 3-D carrier transport simulation framework. We solve the LLT equation numerically with WIAS-pdelib (Fuhrmann et al. 2019), with more details given in O’Donovan et al. (2021b).

To include quantum corrections via LLT into our transport calculations, we make use of the fact that once *u* is determined from solving Eq. (2), an effective potential, *W*, which describes the localization landscape of the confining potential *V*, can be extracted at each mesh-point (Filoche and Mayboroda 2012; Filoche et al. 2017) via:

3 $$\begin{aligned} W(\mathbf {x}_i) = \frac{1}{u(\mathbf {x}_i)} + E_\text {ref}\,\, . \end{aligned}$$

**Fig. 2:**
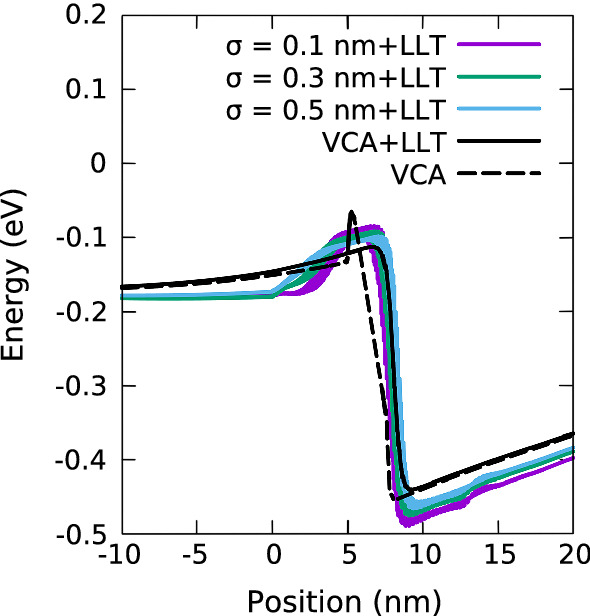
Comparison of valence band edge energies for a In$_{0.1}$Ga$_{0.9}$N single quantum well of width 3.1 nm at 0 V *including* quantum corrections via LLT for a VCA (black, solid) and random alloy calculations using a Gaussian width of 0.1 nm (purple), 0.3 nm (green) and 0.5 nm (blue). The VCA result excluding quantum corrections is also shown (black, dashed). (Color figure online)

To provide first general insight into the impact of LLT corrections to the confining energy landscape for carriers, Fig. 2 shows the effective potential *W* for the VBE of an In$_{0.1}$Ga$_{0.9}$N/GaN SQW system at equilibrium (0 V); the width of the well is 3.1 nm. The data are displayed for three different Gaussian broadening values $\sigma$, namely $\sigma =0.1$ nm (purple), $\sigma =0.3$ nm (green) and $\sigma =0.5$ nm (blue), as well as a LLT corrected VCA profile (black, solid). A “standard” VCA profile is also shown (black, dashed). This figure displays that once LLT is included in the calculations, the impact of $\sigma$ on the band edge profile is significantly reduced. Looking at the VCA plus LLT results, one finds a very smooth confining band edge energy profile. The consequences of using a softened profile for carrier transport will be discussed below.

#### Subtleties of LLT for MQW structures

Before turning to our DD framework and how we use the effective potential *W* in it, we discuss first some subtleties of the LLT approach, which become important when dealing with (In,Ga)N *MQW* systems. To calculate *W*, one has to solve Eq. (2) to obtain *u* first. As discussed in Filoche et al. (2017) and Chaudhuri et al. (2020), *u* can be written as an expansion of the eigenstates $\psi _j(\mathbf {x_i})$ of the system under consideration:

4 $$\begin{aligned} u(\mathbf {x_i}) = \sum _j \alpha _j \psi _j(\mathbf {x_i})\,\, . \end{aligned}$$

The expansion coefficients $\alpha _j$ are then given by

5 $$\begin{aligned} \alpha _j = \sum _{\mathbf {x_i}\in \Omega } \frac{\psi _j(\mathbf {x_i})}{E_j}\,\, . \end{aligned}$$

From Eqs. (4) and (5) it is apparent that *u* and thus the resulting effective potential *W* depends on the magnitude of the energy eigenvalues $E_j$ of a given $\psi _j(\mathbf {x_i})$ and its energetic separation to other (higher lying) states. Thus, if for instance the ground state energy $E_0$ is small (close to 0) and the energy separation to higher lying states $E_j$ with $j\ne 0$ may be large, *u* describes basically the ground state wavefunction (and ground state energy) as one can see from Eq. (4). As a consequence, *u* is a very good approximation of lowest energy state in a given “localization” region $\Omega$. On the other hand, if $E_0$ is large and energetically close to higher lying states, *u* may contain contributions not only from the ground state but also higher lying states. To achieve, on an absolute scale, a small ground state energy one may adjust the energy scale of the system by choosing an appropriate *reference* energy $E_\text {ref}$ such that $E_0-E_\text {ref} > 0$ is small compared to the energy separation with higher lying states. In doing so, *u* and thus the effective potential *W* is dominated by the ground state wavefunction of e.g. an (In,Ga)N QW.

**Fig. 3:**
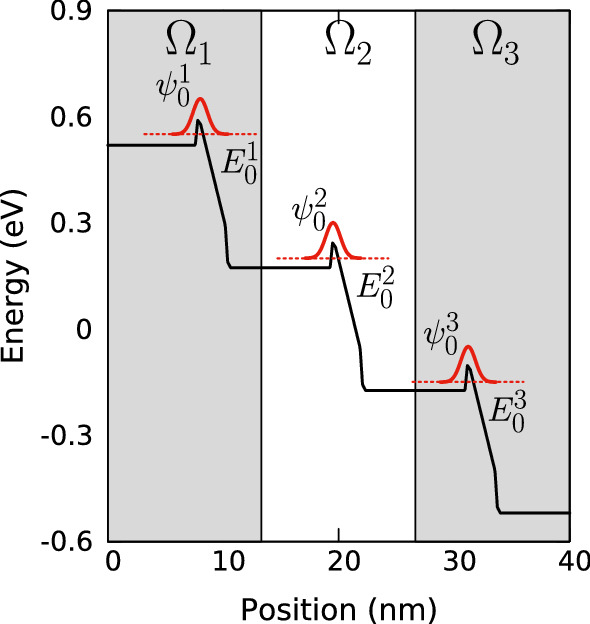
Schematic illustration of a potential band edge energy profile (black solid line) in a multi-quantum well with 3 quantum wells where the wells exhibit a large energy separation between their respective ground state energies $E^i_0$ (red dashed line). The local hole ground state wavefunction in the i$^{th}$ localization region, $\Omega _i$ (marked by shading), are indicated by $\psi ^i_0$ (red, solid). (Color figure online)

While the above can be realised in a straightforward manner for a *SQW* systems (simply using the VBE (CBE) energy as $E_\text {ref}$), for a MQW system this becomes more involved. To illustrate this in more detail, Fig. 3 shows a schematic of a 3 QW system. Here we assume a large energy difference between the VBE values of the different wells to highlight central aspects of LLT. If this structure is treated as one single “localization” region $\Omega$, and we choose the reference energy, $E_\text {ref}$, to be very close to $E_0^1$ (using the hole picture instead of the valence picture), *u* and consequently *W* will be dominated by the ground state wavefunction $\psi ^1_0$, as $\alpha _0\psi _0$ will dominate the series expansion in Eq. (4), originating from Eq. (5). Due to the larger energy separation between $E_\text {ref}$ and $E_0^2$ and $E_0^3$, respectively, there will be basically no contribution from $\psi ^2_0$ and $\psi ^3_0$ to $W(\mathbf {x_i})$. As a consequence the effective potential $W(\mathbf {x_i})$ in the spatial region where $\psi ^2_0$ (located in region $\Omega _2$) and $\psi ^3_0$ (located in region $\Omega _3$) are localized is largely unaffected by LLT quantum corrections.

To circumvent this issues, one could in principle partition the system into multiple (here three) subregions ($\Omega _1,\Omega _2, \Omega _3$) and solve LLT for each sub-system separately; for each subregion an individual $E_\text {ref}^i$ can be chosen. In doing so, the wavefunctions $\psi _0^i$ describe now the ground state wavefunction for each “localization” region $\Omega _i$ with its corresponding local ground state energy $E_0^i$. Now the series expansion of *u* in each region is dominated by the first term, and *u* obtained for each region $\Omega _i$ should give a very good description of the ground state locally. As a consequence, the confining potential in each QW subregion $\Omega _i$ contains quantum corrections.

When using this approach of partitioning the system into different subregions, the remaining question is how to “connect” the local effective potentials $W_i$ so that one obtains a global one, *W*. In the case of electrons, partitioning the system into different localization regions is difficult, as the low effective electron mass leads to a large “leaking” of the wavefunction into the barrier material. This makes it very difficult to connect the individual effective potentials, $W_i$ . Further discussions on consequences of the effective confinement potential for *electron* transport can be found in O’Donovan et al. (2021b). Holes, however, have a much higher effective mass, and partitioning the system is achievable if the separation between the wells in a MQW system is not too small. For the system under consideration (see Sect. 3) this is the case and the locally obtained effective landscapes return quickly to the band edge energy of the GaN barrier material; this guarantees that the interface between neighbouring localization regions is smooth and continuous when “stitching” the different $W_i$ together to obtain *W*. A comparison of effective landscapes obtained with and without partitioning a MQW structure into different sub-regions is show in Appendix A. When analyzing hole carrier transport in a MQW system in Sect. 3.2, we will pay special attention to the above described partitioning of the system when including quantum corrections via LLT in the simulations.

We note also that similar considerations are usually required in “standard” coupled Schrödinger-Poisson simulations of MQW systems. Here, in principle two options are available: solve the Schrödinger equation for a large number of states over the full simulation cell. However, a full quantum mechanical treatment of the full simulation cell is numerically very demanding. Most often, the quantum mechanical description (i.e. solving the Schrödinger equation) of the system is restricted to spatial regions near the wells of a MQW structure with appropriate boundary conditions (e.g. wavefunctions decay approximately to 0 in the barrier material). Such an approach is similar to the above described partitioning of the MQW in different localization regions in which LLT is solved.

### Uni-polar drift-diffusion model

As discussed in Sect. 2.2.1, we transfer the atomistic VBE energy data, together with constant macroscopic VBE parameters for the doped regions, on to a FVM mesh. Following the discussion in the previous section, we may use for the atomistic VBE data either $E_v^{\sigma }(\mathbf {x}_i)$, see Eq. (1), or $-W(\mathbf {x}_i)$, see Eq. (3); the multiplication of *W* by $-1$ is due to the change from the hole picture to the valence band picture. Next, we present the DD models which describe charge transport through our device.

Charge carrier transport is modelled using the van Roosbroeck system (Van Roosbroeck 1950). As we are interested in uni-polar hole transport, the stationary van Roosbroeck system consists of two coupled nonlinear partial differential equations of the form:

6a $$\begin{aligned}&-\nabla \cdot \left( \varepsilon _s(\mathbf {x}) \nabla \psi (\mathbf {x})\right) =q\left( p(\mathbf {x}) + C\right) \,\, , \end{aligned}$$

6b $$\begin{aligned}&\nabla \cdot \mathbf {j}_p = 0 \end{aligned}$$

for $\mathbf {x} \in \Omega$. The Poisson equation, Eq. (6a), describes the electric field generated by the scalar electric potential $\psi (\mathbf {x})$ in the presence of a free (hole) charge carrier density, $p(\mathbf {x})$. Here, $\varepsilon _s(\mathbf {x}) = \varepsilon _0\varepsilon _r(\mathbf {x})$ describes the position dependent dielectric constant; *q* is the elementary charge. In a (doped) uni-polar semiconductor device, the overall charge density is given by the density of free (positively charged) holes, $p(\mathbf {x)}$ and the density of singly ionized acceptor atoms $N_A^+(\mathbf {x})$ (where $C=-N_A^+(\mathbf {x})$). Intrinsic electrostatic built-in fields, arising from spontaneous and piezoelectric polarization, are directly included in the confining energy landscape generated from TB. Thus, band edge energies contain, in addition to macroscopic effects, also local polarization fluctuations (via a local polarization theory, discussed in Caro et al. (2013)) that stem from alloy fluctuations. Consequently, (spontaneous and piezoelectric) polarization charges are treated on an atomistic level and enter the Poisson equation through band edge profile. The current density $\mathbf {j}_p(\mathbf {x})$ is given by (Farrell et al. 2017)

7 $$\begin{aligned} \mathbf {j}_p(\mathbf {x}) = -q\mu _p p(\mathbf {x}) \nabla \varphi _p(\mathbf {x})\,\, . \end{aligned}$$

That is, the negative gradient of the quasi Fermi potential, $\varphi _p(\mathbf {x})$, is the driving force of the current; $\mu _p(\mathbf {x})$ denotes the free carrier (hole) mobility.

To describe the density of free carriers, $p(\mathbf {x})$, in a solid one can either use Boltzmann or Fermi-Dirac statistics. In the following we employ the Boltzmann approximation, but provide a short discussion on the impact of the Fermi-Dirac distribution function on the results in Appendix B. Using the Boltzmann approximation $p(\mathbf {x})$ is given by

8 $$\begin{aligned} p(\mathbf {x})&= N_v \exp \left( \frac{q\left( \varphi _p\left( \mathbf {x}\right) - \psi \left( \mathbf {x}\right) \right) + E_v^{dd}(\mathbf {x})}{k_B T} \right) , \end{aligned}$$

where $k_B$ is the Boltzmann constant, *T* denotes the temperature, $E_v^{dd}(\mathbf {x})$ is the (position dependent) VBE energy and $N_v$ is the effective density of states:

$$\begin{aligned} N_v = 2\Bigg (\frac{2\pi m^*_h k_B T}{\hbar ^2}\Bigg )^{3/2}. \end{aligned}$$

The VBE energy $E_v^{dd}$ in the DD simulations may now be chosen to be (smoothed) TB data, $E_v^{dd} = E_v^{\sigma }$, VCA data, $E_v^{dd} = E_v^\text {VCA}$, or the outcome of the LLT calculations, $E_v^{dd} = -W$. In doing so, the VBE energy, $E_v^{dd}$, may vary spatially due to random alloy fluctuations. Thus, care must be taken to discretize the hole flux correctly. To this end we extend the well-known Scharfetter-Gummel flux approximation (Scharfetter and Gummel 1969) to variable band edge energy values, as detailed in O’Donovan et al. (2021). Bias values are implemented via Dirichlet boundary conditions. Details of this approach can be found in Farrell et al. (2017).

## Results

**Table 1:** Material parameters used in the simulations

Physical quantity	Value	Units
$m_h^\star$ GaN	1.87	m$_0$
$m_h^\star$ InN	1.61	m$_0$
$\mu _h\ p-$GaN	5	cm$^2$/(V s)
$\mu _h\ i-$GaN	10$^\dagger$	cm$^2$/(V s)
$\mu _h\ i-$(In,Ga)N	10	cm$^2$/(V s)
$\epsilon _r^\text {GaN}$	9.7	$\epsilon _0$
$\epsilon _r^\text {InN}$	15.3	$\epsilon _0$
$p-$doping (GaN)	2$\times$10$^{19}$	cm$^{-3}$

Unless otherwise specified, all parameters are taken from Li et al. (2017);

$^\dagger$ Li et al. (2014)

**Fig. 4:**
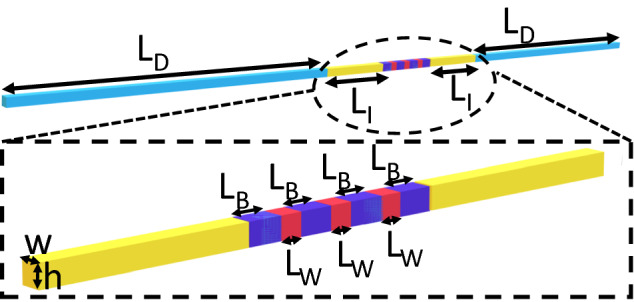
Schematic illustration of the simulation cell with three quantum wells (QWs) in the active region. The *p*-doped regions (light blue) have a doping density of $2\times 10^{19}$ cm$^{-3}$ and a length of $L_D =$ 160 nm. The intrinsic regions on the coarse mesh (yellow) have a length of $L_I = 40$ nm. The atomistic region, also assumed as intrinsic, contains regions of a GaN barrier material (dark blue) with a length of $L_B =8.0$ nm and In$_{0.1}$Ga$_{0.9}$N QWs (red) with a width of $L_W =3.1$ nm. For a single QW calculation the atomistic region contains only one In$_{0.1}$Ga$_{0.9}$N QW ($L_w=3.1$ nm) and two GaN barrier regions. The simulation cell has an in-plane dimension of $w \times h =5.1 \times 4.4$ nm$^2$ along the entire system. (Color figure online)

In this section, we apply the framework described above to a *p*-doped-intrinsic-*p*-doped (*p*-*i*-*p*) system in both a SQW, Sect. 3.1, and a MQW, Sect. 3.2, setting. Our simulations are carried out within the ddfermi simulation tool (Doan et al. 2020), which is implemented within the WIAS-pdelib toolbox (Fuhrmann et al. 2019). A schematic of the MQW system including the contact regions is shown in Fig. 4. Details about well and barrier widths, as well as the In content are given in the figure caption. The material parameters entering the DD calculations are summarized in Table 1; in all calculations the temperature *T* is set to $T=300$ K. To study the influence of alloy fluctuations and quantum corrections on the carrier transport, the simulations have been performed for the different $E_v^{dd}$ settings discussed in Sect. 2.3. Thus, we compare results from calculations including alloy fluctuations to results from VCA simulations; the simulations have been carried out in the absence and presence of LLT quantum corrections. In the case of the MQW, we also investigate how the current-voltage (I–V) curves change when partitioning the MQW system to solve LLT locally (for each QW), see also discussion in Sect. 2.2.4.

### Single QW

**Fig. 5:**
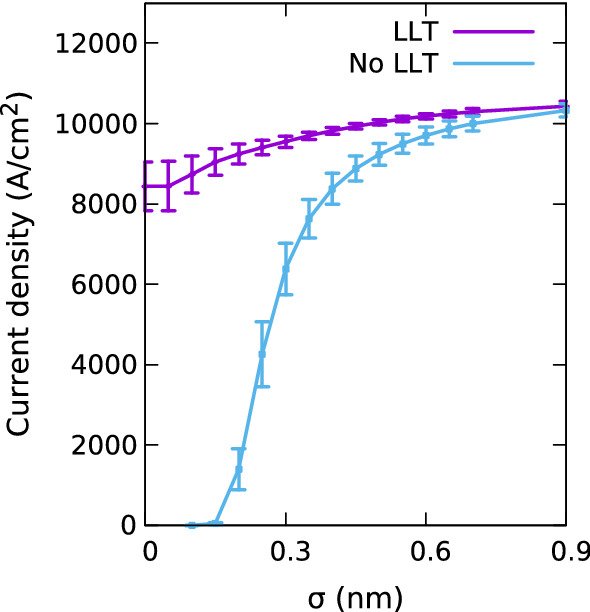
Impact of Gaussian width, $\sigma$, on the current in a single In$_{0.1}$Ga$_{0.9}$N/GaN quantum well system at a bias of 1.0 V. Results are obtained in the presence (purple) and absence (blue) of quantum corrections via LLT and are averaged over 5 different microscopic configurations. The errorbars show the standard deviation of the current over the 5 configurations. (Color figure online)

In the following we analyze the impact of random alloy fluctuations and quantum corrections on the I–V characteristics of a *p*-*i*-*p* (In,Ga)N SQW system; details of the structure and simulation cell are given in the caption of Fig. 4. In order to study the influence of the alloy microstructure on the results we have repeated these calculations for 5 different microscopic configurations. Furthermore, the Gaussian broadening $\sigma$ has been varied to study how $\sigma$ affects the results. Before turning our attention to the full I–V curve of the system, Fig. 5 depicts the current in the SQW system at a fixed bias of 1.0 V for different $\sigma$ values. As discussed in Sect. 2.2, when $\sigma$ is increased, the Gaussian function softens the VBE and reduces the magnitude of the fluctuations. As consequence, in the *absence* of quantum corrections, the current at 1.0 V increases with increasing $\sigma$ and starts to converge for $\sigma$ values larger than approximately 0.5 nm. For these large $\sigma$ values the VBE becomes smooth and the current approaches that of a VCA without quantum corrections, as we will discuss further below. In addition, Fig. 5 also reveals that there is an abrupt increase in the current at around $\sigma =0.2$ nm. We attribute this to the fact that if $\sigma$ is small and below the bond length of e.g. GaN, the band edge profile entering the DD simulations exhibits strong (local) fluctuations which noticeably affect the carrier transport.

**Fig. 6:**
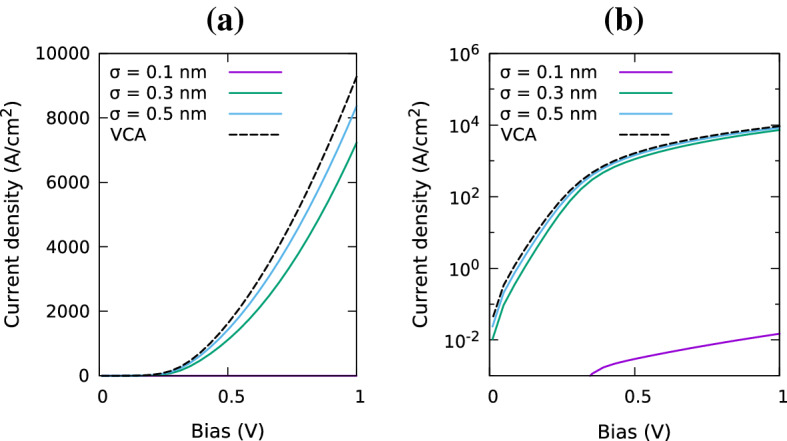
Comparison of current-voltage curves for a single In$_{0.1}$Ga$_{0.9}$N/GaN quantum well for VCA (black, dashed) and random alloy calculations using a Gaussian width of $\sigma =0.1$ nm (purple), $\sigma =0.3$ nm (green) and $\sigma =0.5$ nm (blue) in the absence of quantum corrections. Results are shown on a linear scale (left) and log scale (right). (Color figure online)

In the next step we turn our attention to the full I–V curves in the presence of alloy fluctuations but the *absence* of LLT quantum corrections. Overall, the behavior discussed for the fixed bias of 1.0 V, Fig. 5, is also reflected in the full I–V curves, Fig. 6: for a Gaussian width of $\sigma = 0.1$ nm the current is extremely low, but increases with increasing $\sigma$. However, it is important to note that the here obtained results are in contrast to uni-polar *electron* transport, for instance discussed in O’Donovan et al. (2021b). In the case of the electrons, the current always *exceeds* the VCA results, while we find here that in the hole case it *approaches* the VCA data. This means that for electron transport alloy fluctuations are beneficial, while they are detrimental for the hole transport in (In,Ga)N QWs. This result is consistent with the observation that alloy fluctuations lead to strong hole localization effects, while electron wavefunctions, due to their lower effective mass, are affected to a lesser extent by the alloy fluctuations (Watson-Parris et al. 2011; Schulz et al. 2015).

**Fig. 7:**
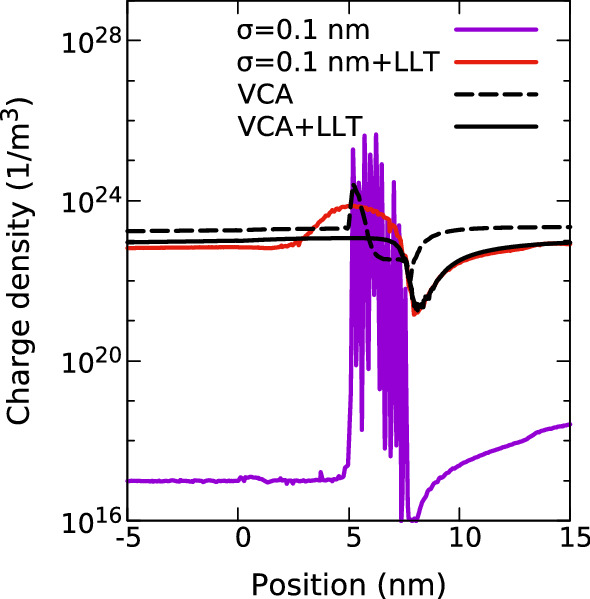
Carrier density distribution in and around a single In$_{0.1}$Ga$_{0.9}$N/GaN quantum well of width 3.1 nm at a bias of 1.0 V for calculations including random alloy fluctuations and using a Gaussian width of 0.1 nm. The results are shown in the absence (purple) and presence (red) of quantum corrections via LLT. For comparison VCA data (black, dashed), and VCA including LLT (black, solid) are also depicted. (Color figure online)

To shed more light onto the influence of alloy fluctuations on the hole transport, Fig. 7 shows the charge density distribution in and around the (In,Ga)N SQW region for $\sigma = 0.1$ nm in the absence of any LLT quantum corrections and at a bias of 1.0 V. For comparison the VCA charge density distribution is depicted (black, dashed) as well as the VCA charge density distribution including quantum corrections (black, solid). We stress again that due to the small $\sigma$ value, the alloy fluctuations lead to a strongly fluctuating VBE energy profile, which in turn results in strong hole localization effects. From Fig. 7 one can infer that due to the strong carrier localization effect, the carrier density is very high when compared to the VCA result in the QW region; the carrier density in the barrier material is depleted in the random alloy case compared to VCA. As a consequence, these carrier localization effects/the strong VBE fluctuations lead to a strong VBE bending, originating from the coupling of the hole density and the quasi-Fermi level via Eqs. (7) and (8). Overall, and compared to the VCA result, this gives rise to a larger resistivity of the device. Thus for this small value of $\sigma =0.1$ nm, the current through the device is very low, as seen in Fig. 6. We note that such a low broadening parameter can result in an underlying energy landscape which is not compatible with the DD framework (as $\sigma$ is much smaller than the de Broglie wavelength), and this extreme depletion of the barriers may be physically unrealistic.

**Fig. 8:**
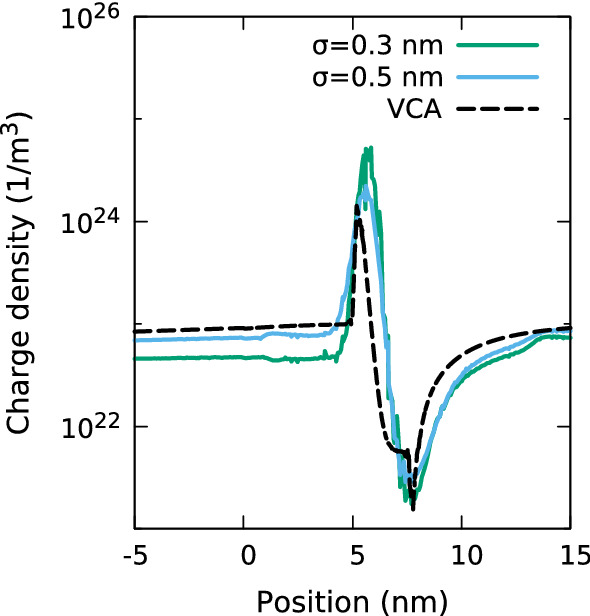
Carrier density distribution in and around a single In$_{0.1}$Ga$_{0.9}$N/GaN quantum well of width 3.1 nm at a bias of 1.0 V for a VCA (black, dashed) and random alloy calculations. The latter use Gaussian widths of $\sigma =0.3$ nm (green) and $\sigma =0.5$ nm (blue) and exclude quantum corrections. (Color figure online)

The situation changes with increasing $\sigma$ as Fig. 8 shows. Here, the charge density distribution in and around the QW for both $\sigma = 0.3$ nm (green) and $\sigma = 0.5$ nm (blue) are similar to the VCA results (black, dashed). Furthermore, as the charge density distributions with increasing $\sigma$ approaches the VCA profile, so does the resulting I–V curve, Fig. 6.

**Fig. 9:**
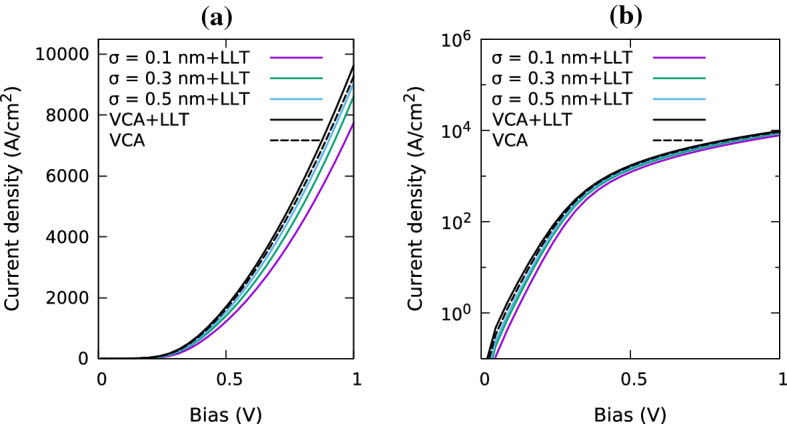
Including quantum corrections via LLT: Comparison of current-voltage curves for a single In$_{0.1}$Ga$_{0.9}$N/GaN quantum well of width 3.1 nm for a VCA (black, solid) and random alloy calculations; the random alloy simulations use Gaussian widths of $\sigma =0.1$ nm (purple), $\sigma =0.3$ nm (green) and $\sigma =0.5$ nm (blue). Results are shown on a linear scale (left) and log scale (right). (Color figure online)

Having discussed the impact of alloy fluctuations on the hole transport, we focus our attention now on the impact of quantum corrections on the results. Overall, we find that when including quantum corrections via LLT in the transport calculations, the Gaussian width $\sigma$ influences the results to a much lesser extent. This can for instance been seen in Fig. 5, where the current is shown as a function of $\sigma$ (purple line) at a fixed bias of 1.0 V. In contrast to the results without quantum corrections (light blue), when including these corrections, the obtained current changes very little when increasing $\sigma$ beyond 0.2 nm. We highlight also that even at the very low $\sigma$ value of $\sigma =0.1$ nm, the current is strongly increased when including quantum corrections. The origin of this becomes clear when looking again at the carrier density profile in and around the SQW, depicted in Fig. 7. As discussed above, in the absence of quantum corrections, the strongly fluctuating energy landscape leads to a very large carrier density in the well and depletes the region surrounding the well. When accounting for quantum corrections, the carrier density profile including alloy fluctuations (red), even though the same $\sigma$ value is used, is much smoother and approaches the VCA quickly in the barrier. This emphasizes again that quantum corrections soften the confining energy landscape and indicates also that once LLT corrections are taken into account, the importance of alloy microstructure is reduced. This is further supported by Fig. 5: the standard deviation (indicated by the error bars in the figure) is small relative to the current, at least for larger $\sigma$. The impact of the alloy microstructure is still visible for smaller $\sigma$ values. We note here also that the magnitude of this effect may depend on the in-plane dimension of the simulation cell, especially when using small $\sigma$ values. Thus careful studies are required to analyze this in more detail, including a further evaluation on the choice of the “correct” Gaussian width before LLT is applied. The impact of the in-plane dimension on current density in a SQW is further discussed in Appendix C.

When turning to the full I–V curve of the SQW system, Fig. 9, we find that the choice of $\sigma$ is of secondary importance, at least for the system studied here. In addition to the random alloy calculations, Fig. 9 depicts also VCA results both in the presence (black, solid) and absence (black, dashed) of LLT quantum corrections. From this it is clear that in the case of a SQW, random alloy results do not differ strongly from the VCA data. Interestingly, these results are also well approximated by VCA simulations *excluding* quantum corrections. For the VCA, when there are no alloy fluctuations and the VBE is smooth, the combination of the small valence band offset as well as the high hole effective mass, results in similar profiles for the confining potentials of the VCA and quantum corrected VCA. Consequently the I–V curves do not differ significantly.

It should be noted that the above discussed results are different but also similar to uni-polar *electron* transport. They are similar in the sense that once quantum corrections are taken into account, VCA and random alloy simulations give very similar results in terms of the I–V characteristics of SQW systems. However, a difference between electron and hole transport is that for uni-polar electron transport the current increases for larger $\sigma$ values and exceeds the VCA result, for holes this is not the case. Our calculations also indicate that for holes, once LLT corrections are included, the current is not strongly dependent of $\sigma$. However, it should again be noted that this result may depend on the in-plane dimensions of the simulation cell. A larger in-plane cell may give rise to a larger extent of locally varying band edge energies. As a consequence carrier localization effects may be more pronounced. Thus the here presented results should be treated as “best” case scenario, since when carriers are “trapped” by alloy fluctuations they will increase the resistivity of the device. We conclude therefore that in general carrier localization effects will have a detrimental effect on the hole transport, and the resulting currents will in general be smaller or equal to the VCA result, in contrast to electrons.

But, the impact of carrier localization effects on the I–V curves may be more pronounced in MQWs, as the depletion of the carriers in the GaN barrier region may be amplified in such a system when compared to a SQW. In our previous study on uni-polar *electron* transport we have already seen that results from a SQW system cannot necessarily be carried over to MQWs. In general, gaining insight into hole transport in MQW systems is very important for understanding the carrier distribution in full (In,Ga)N-based MQW LED structures. Thus, we turn our attention in the next section to uni-polar hole transport in (In,Ga)N MQW structures.

### Multi QW

**Fig. 10:**
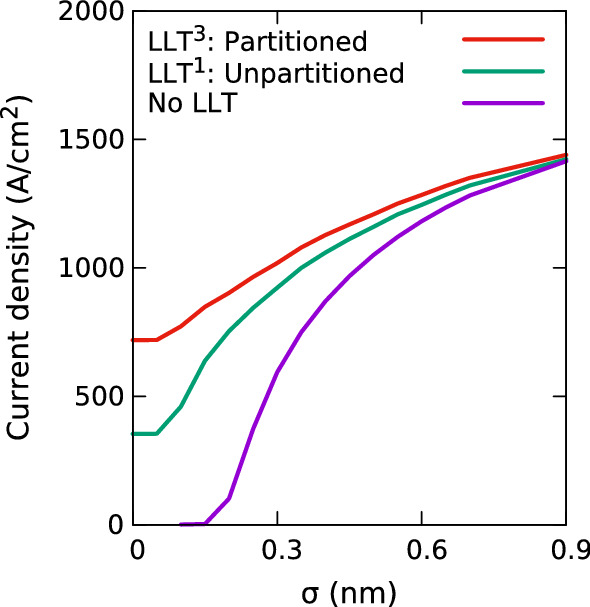
Impact of Gaussian width, $\sigma$, on the current in an In$_{0.1}$Ga$_{0.9}$N/GaN multi-quantum well system at 1.0 V. Results are shown (i) for a system including quantum corrections via LLT and partitioning the system into 3 localization regions each with a local reference energy (LLT^3^, red), (ii) for a system including quantum corrections via LLT using a single (global) reference energy for the entire multi-quantum well region (LLT^1^, green), and (iii) for a system excluding quantum corrections (No LLT, purple). (Color figure online)

Similar to the SQW system discussed in the previous section, we start our analysis of the uni-polar hole transport in a (In,Ga)N/GaN MQW system by investigating the impact of the Gaussian width $\sigma$ on the results. Figure 10 displays the current through the MQW system as a function of $\sigma$ at a fixed bias of 1.0 V. Here we compare results from simulations that (i) exclude quantum corrections via LLT (purple), (ii) include quantum corrections via LLT but treating the entire MQW region as one localization region (green), and (iii) quantum corrections via LLT but solving the LLT equation for each well of the MQW system separately (red), as discussed in Sect. 2.2 (cf. Fig. 3).

Figure 10 shows that for all studied $\sigma$ values, the calculation excluding LLT (purple line) exhibits the lowest current at a fixed voltage of 1.0 V. Also, the difference is largest at small $\sigma$ values. In the case of the calculation without LLT corrections, the VBE exhibits large local fluctuations. These fluctuations are intrinsically smoothed by the quantum corrections, and the resulting landscape (even for small $\sigma$ values) exhibits significantly smaller fluctuations due to the alloy microstructure. The large VBE fluctuations increase the potential barrier and consequently increase the resistance in the *p*-*i*-*p* junction, thus leading to a smaller current. This is the same effect we have already seen in the SQW system, however, the effect is more pronounced due to the combined influence of the 3 QWs in the MQW.

In a second step we discuss the results from the calculations including quantum corrections in more detail. Looking at the simulations using a global reference energy, i.e. the MQW system is treated as a single localization region (green), we find that the current drops a greater amount at low $\sigma$ values compared the the outcome of the simulations using a local reference energy (where each well is treated as a separate localization region). More specifically, at the smallest considered $\sigma$ value (no broadening), the current obtained from the model using a global reference energy is just over half the current using local reference energies. We attribute this drop to the combination of two factors. Firstly, given that the LLT model using a local reference energy also shows a slight drop in current with decreasing $\sigma$ indicates that the strong fluctuations in the VBE energy still impact the current even though the LLT treatment softens this intrinsically. Secondly, when treating the MQW as a single localization region, a poorer description of the confining potential of the QW for which the VBE energy is furthest away from the global reference energy is expected in such an LLT treatment. As a consequence, still larger fluctuations are present in the wells energetically furthest away from the reference energy, especially for small $\sigma$ values. All this will result in a higher resistivity of the MQW system and consequently a lower current at a fixed bias.

**Fig. 11:**
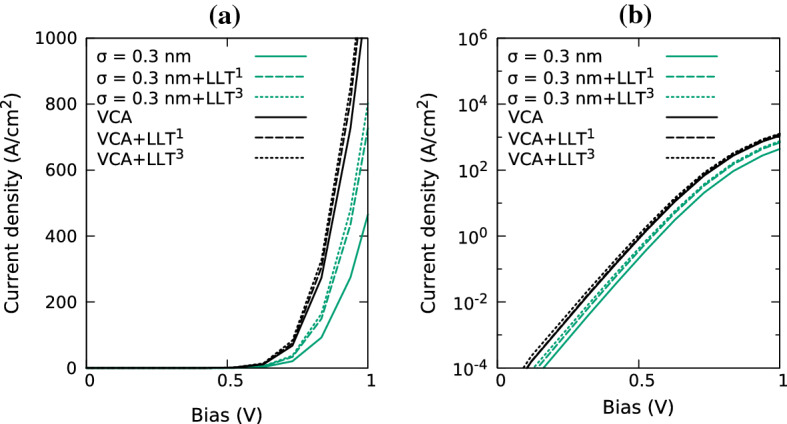
Comparison of current-voltage curves in a multi-quantum well In$_{0.1}$Ga$_{0.9}$N/GaN system for VCA (black) and random alloy calculations; the random alloy simulations use a Gaussian width of 0.3 nm (green). I–V curves are shown for calculations without any quantum corrections (solid), including quantum corrections when employing an un-partitioned (dashed, superscript ‘1’) and partitioned multi-quantum well regions (dotted, superscript ‘3’). Results are shown on (**a**) a linear scale and (**b**) a log scale. (Color figure online)

Having discussed the impact of Gaussian broadening and LLT quantum corrections on the current in a MQW system at a fixed bias, Fig. 11 depicts the full I–V curves. Here again results from calculations applying LLT, both using a single localization region (dashed), $\Omega$, and sub-regions, $\Omega _i$, for each QW (dotted), as well as results in the *absence* of quantum corrections (solid) are shown. This is displayed for both VCA (black) and random alloy calculations using a Gaussian width of 0.3 nm (green); to get first insight into the hole transport in a MQW structure we have restricted the calculations to one alloy configuration. Future studies can target analysing the statistics of different alloy microstructure configurations on the results. A value of $\sigma = 0.3$ nm has been chosen since it is large enough for the Gaussian averaging to including neighbouring sites but small enough to still capture effects due to carrier localization. Figure 11 reveals that in both VCA and random alloy calculations, quantum corrections increase the current similar to the situation in uni-polar electron transport (O’Donovan et al. 2021b). Furthermore, when using a local reference energy for LLT, thus treating each QW as an individual localization region, $\Omega _i$, the current increases further when compared to the LLT model employing a global reference energy. Our results also show that this effect is more pronounced for the random alloy case; partitioning the system in VCA impacts the I–V curve (black dashed and black dotted line) only slightly.

Overall our calculations reveal that in the MQW system and for the chosen $\sigma$ value of $\sigma =0.3$ nm, even when including LLT corrections, the random alloy calculations give a smaller current at fixed bias when compared to the VCA result. This finding is in contrast to the SQW system, where VCA and random alloy results give very similar results, see Fig. 9. Furthermore, and again in contrast to the SQW structure, the magnitude of the difference in current between VCA and random alloy results will depend on the $\sigma$ value, as Fig. 10 shows. Future studies targeting for instance theory experiment comparisons are now required to gain further insight into the broadening parameter $\sigma$. We note that beyond $\sigma$, and as already mentioned above, the in-plane dimension of the simulation cell may impact the results as carrier localization effects due to *lateral* fluctuations in the (In,Ga)N wells can have a (detrimental) influence on the current. Furthermore, it should be noted that the LLT treatment builds on a single-band effective mass approximation; our previous studies indicate that such a model may underestimate hole localization effects (Chaudhuri et al. 2021), which in turn may lead to higher current.

Nevertheless, we would expect that all these factors only reduce the current further in the MQW system. Thus the VCA I–V curve should be regarded as a upper bound for the hole current in an (In,Ga)N MQW structure. This is in contrast to uni-polar electron transport, where alloy fluctuations and quantum corrections give rise to an *increase* in the current when compared to a VCA result (O’Donovan et al. 2021b). Overall, we conclude that alloy disorder has a *detrimental* effect on *hole* transport (In,Ga)N MQWs. The degree to which this impacts the I–V curve requires further careful research into the description of the confining energy landscape.

## Conclusion

In this work we applied the previously established TB-to-continuum framework to perform drift-diffusion calculations for *p*-*i*-*p* systems. The impact of alloy fluctuations was determined by comparing to a VCA, and quantum corrections were included via LLT. Our results showed that alloy fluctuations have a detrimental effect on hole transport through In$_{0.1}$Ga$_{0.9}$N/GaN QW systems, although the degree to which this impacts results depends on the treatment of the localization landscape, and the smoothing applied. For low Gaussian broadening values, $\sigma$, the alloy fluctuations reduce the current, due to the increased hole density localizing within the QWs and the resulting depletion of the barriers; this reduces the conductivity in the barrier regions. When the landscape is heavily smoothed (large $\sigma$) this effect is reduced, and the I–V curve approaches that of VCA (a smooth landscape). As already highlighted above, further studies on how to describe the (disordered) energy landscape are now required to shed more light onto the carrier transport in (In,Ga)N/GaN QW systems.

## Figures and Tables

**Fig. 12:**
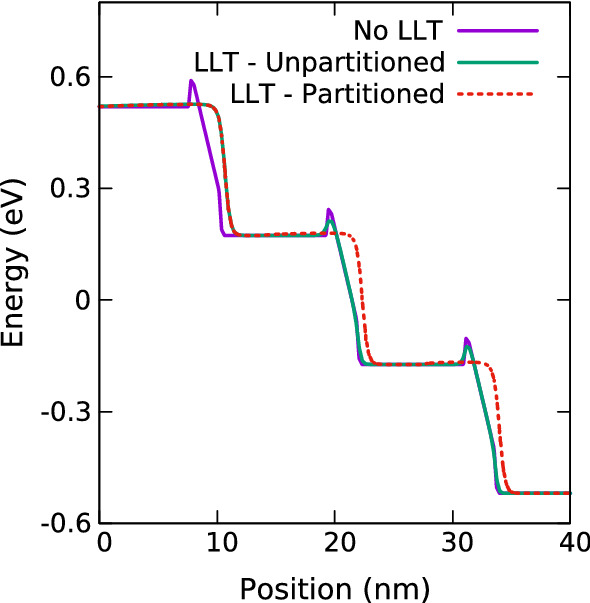
Valence band edge profile in virtual crystal approximation for a fictive (In,Ga)N/GaN multi-quantum well system in the absence of LLT (purple) and presence of LLT quantum corrections. When including LLT two scenarios are considered (i) using a single reference energy (green) and (ii) separate reference energies for each quantum well region (red, dashed). (Color figure online)

**Fig. 13:**
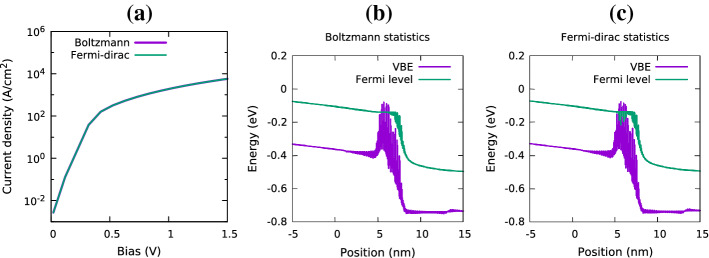
Impact of distribution function for a random alloy system using a Gaussian width of $\sigma = 0.2$ nm on the I–V curve, the valence band edge and Fermi-level in the (In,Ga)N/GaN SQW described in the main text. Left: I–V curves for Boltzmann (purple) and Fermi-Dirac (green). Center: Valence band edge (purple) and Fermi level (green) at 0.5 V using Boltzmann statistics. Right: Valence band edge (purple) and Fermi level (green) at 0.5 V using Fermi-Dirac statistics. (Color figure online)

**Fig. 14:**
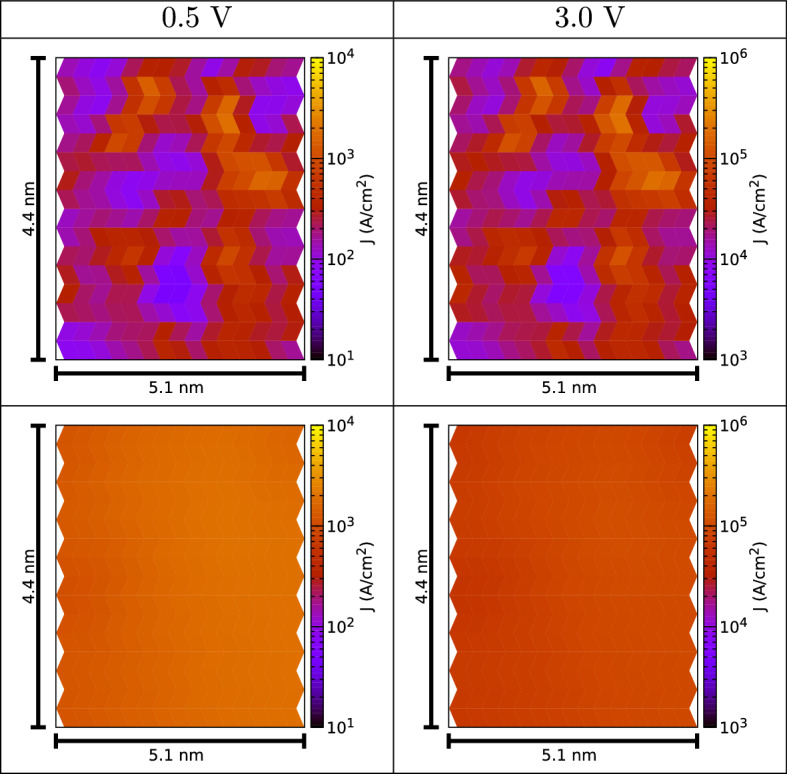
Current density within a slice of a (In,Ga)N SQW at 0.5 V (left) and 3.0 V (right) using a Gaussian broadening of 0.2 nm without LLT (top) and with LLT (bottom). The total current density is shown using the colour scale on a log scale. (Color figure online)

## Appendix A: Effective confining potential in MQW structure: partitioned vs unpartitioned LLT

In this appendix we provide further insight into the question how the effective confining potential, *W*, obtained from LLT is modified when partitioning the MQW into sub-regions, i.e. different “localization” regions. As discussed in Sect. 2.2.4, the choice of the reference energy $E_\text {ref}$ for a given localization region can impact the resulting quantum corrected effective landscape. As a test case we use the system discussed in the main part of the manuscript which exhibits a large potential difference between the QWs (as shown in Fig. 3) forming the MQW. For demonstrative purposes we neglect any effects due to the presence of a *p*-*i*-*p* junction and we assume a capacitor-like potential profile with a potential drop across each QW of 0.35 V. Figure 12 reveals the impact that partitioning the MQW into different subregions has on the effective band edge. The starting point is the “standard” VCA description of the system without quantum corrections (purple). Here, each QW exhibits the same VBE profile. Treating the MQW system as a single localization region within LLT, the resulting profile (green) reveals that the band edge of the first QW (leftmost in Fig. 12) is smoothed significantly. However, the two other wells forming the MQW system, which are energetically far from the global reference energy chosen, undergo noticeably smaller corrections. As discussed in the main text, this stems from the fact that the contributions from (localized) states in these QWs contribute only weakly to the series expansion of *u* (Eqs. (4) and (5)). However, Fig. 12 also reveals that when the system is partitioned into 3 sub-regions, and each localization region (QW region) is described by an individual reference energy which is close the *local* ground state energy, the resulting effective landscape (red, dashed) is significantly softened in all three wells of the MQW system. In doing so, one assures that quantum corrections in all 3 QWs are properly treated. Figure 12 also confirms that the landscape is not only smoothed but also continuous between each localization region, which is important to construct a global effective landscape that can be used for transport calculations.

## Appendix B: Comparison of impact of distribution function on results

The results presented in the main part of the manuscript rely on the Boltzmann approximation for free carrier density. In this appendix we briefly discuss how and if the results are affected by employing Fermi-Dirac statistics instead of Boltzmann. Overall, we find that in the case of the here studied uni-polar hole transport, the resulting I–V curves are basically unaffected when changing the distribution function from Boltzmann to Fermi-Dirac. This is illustrated in Fig. 13 (a) for a SQW.

To shed more light onto this finding, we have also investigated how the Fermi-level changes when using Fermi-Dirac instead of Boltzmann. The resulting Fermi-levels (green) for the two distribution functions are depicted in Fig. 13b (Boltzmann) and c (Fermi-Dirac). In addition to the Fermi-level, the valence band edge (purple) is also given. The data are plotted at 0.5 V, for a relatively low Gaussian width of $\sigma = 0.2$ nm and when excluding localization landscape theory (LLT). Comparing the Fig. 13b (Boltzmann) and c (Fermi-Dirac), one observes that choosing Fermi-Dirac statistics only very weakly affects the Fermi-level inside the well; in the barrier it is basically unchanged. This explains our finding that both distribution functions give basically the same I–V curve. Above we used a calculation without LLT and a relatively low $\sigma$ value ($\sigma =0.2$ nm), so that differences between Boltzmann and Fermi-Dirac will be further reduced when using a larger broadening (larger $\sigma$ value) and/or when including LLT in the calculations, since both contributions will result in a smoother energy landscape (not shown).

## Study of the in-plane dimensions on current density

In the main text we have already highlighted that the results may depend on the in-plane dimension of the simulation cell. Here, we extend this discussion. Figure 14 depicts the current density for an in-plane slice through the an In$_{0.1}$Ga$_{0.9}$N SQW for two different applied voltages, namely 0.5 V (left column) and 3.0 V (right column); for the Gaussian broadening we have used $\sigma =0.2$ nm. The upper row displays the data in the absence of LLT corrections, while the lower row depicts the results in the presence of LLT corrections.

Looking at the results *without* LLT, one can clearly see that that current density strongly fluctuates within the plane. This means that in *the absence* of quantum corrections via LLT, the simulation cell is large enough to resolve the alloy fluctuations and potentially connected percolation paths. In the main text we have compared I–V curves without LLT but in the presence of alloy fluctuations to VCA results, obtained also in the absence of LLT. We observed that the turn on voltage/knee voltage for the hole transport is higher and the current is lower in the alloy case when compared to the VCA. Therefore, the observed fluctuations in the current density have detrimental impact on the hole transport. Given that we resolve the variation in the current density already for the present simulation cell size, one could expect that the observed results will not change dramatically when increasing the in-plane dimension of the simulation cell.

However, when including LLT in the calculations, the alloy fluctuations are “washed out” for the given cell size as the lower row of Fig. 14 shows. Here, the current density indeed behaves more like the VCA case. But, if the in-plane dimension of the simulation cell is increased, the softening of the energy landscape will be reduced, as the likelihood of regions with locally high/low indium content increases. Thus we can expect to recover an in-plane current density profile with regions of high and low current, similar to the calculation neglecting quantum corrections, but on a larger scale. As a consequence, with increasing simulation cell size and even with LLT, it is expected that one finds a situation more similar to the results in the absence of LLT, thus the upper row in Fig. 14. As a consequence it is expected that in case of LLT and random alloy fluctuations, the current density decreases with increasing in-plane dimension. As such, our conclusion in the paper that the VCA I–V curve presents an upper limit for the hole transport, should still hold even when increasing the in-plane dimensions of the simulation cell.

## Footnotes

1 As the derivation of LLT requires that $\hat{H}^\text {EMA}$ is a positive definite operator (Filoche and Mayboroda 2012), Eq. (2) is solved in the hole picture (where the hole ground state has the *lowest* energy on an absolute scale, and the hole effective mass is *positive*) rather than in the valence band picture (where the hole ground state has the *highest* valence band energy on an absolute scale, and the hole effective mass is *negative*). As such, the confining potential is described by $V = -E^\text {TB}_v$. The resulting effective landscape, *W* (Eq. 3), is converted to the valence band picture (multiplication by -1) so that it can be used in transport calculations. *When displaying band edge profiles, we always use the valence band picture; if LLT has been applied, the obtained effective landscapes/potentials, W, have been transformed accordingly.*
